# Psychosocial Aspects of Gestational Grief in Women Undergoing Infertility Treatment: A Systematic Review of Qualitative and Quantitative Evidence

**DOI:** 10.3390/ijerph182413143

**Published:** 2021-12-13

**Authors:** Michelle Herminia Mesquita de Castro, Carolina Rodrigues Mendonça, Matias Noll, Fernanda Sardinha de Abreu Tacon, Waldemar Naves do Amaral

**Affiliations:** 1 Postgraduate Program in Health Sciences, Faculdade de Medicina, Universidade Federal de Goiás, Goiânia 74605-050, Brazil; matias.noll@ifgoiano.edu.br (M.N.); fernandabreu2010@yahoo.com.br (F.S.d.A.T.); dr@waldemar.med.br (W.N.d.A.); 2 Campus Ceres, Instituto Federal Goiano, Ceres 76300-000, Brazil; 3 Department of Sports Science and Clinical Biomechanics, University of Southern Denmark, 5230 Odense, Denmark

**Keywords:** grief, fetal death, abortion, spontaneous, psychological wellbeing, infertility

## Abstract

Women undergoing assisted reproduction treatment without being able to become pregnant, and experiencing pregnancy loss after assisted reproduction, are triggering factors for prolonged grief and mourning. This review aims to investigate the psychosocial aspects of gestational grief among women who have undergone infertility treatment. We searched the databases of MEDLINE/PubMed, EMBASE, CINAHL, Scopus, ScienceDirect, and Lilacs for works published up to 5 March 2021. The outcomes analyzed were negative and positive psychosocial responses to gestational grief among women suffering from infertility and undergoing assisted human reproduction treatment. Eleven studies were included, which yielded 316 women experiencing infertility who were undergoing treatment. The most frequently reported negative psychosocial manifestations of grief response were depression (6/11, 54.5%), despair or loss of hope/guilt/anger (5/11, 45.5%), anxiety (4/11, 36.4%), frustration (3/11, 27.3%), and anguish/shock/suicidal thoughts/isolation (2/11, 18.2%). Positive psychosocial manifestations included the hope of becoming pregnant (4/6, 66.6%) and acceptance of infertility after attempting infertility treatment (2/6, 33.3%). We identified several negative and positive psychosocial responses to gestational grief in women experiencing infertility. Psychological support before, during, and after assisted human reproduction treatment is crucial for the management of psychosocial aspects that characterize the grief process of women experiencing infertility who become pregnant and who lose their pregnancy. Our results may help raise awareness of the area of grief among infertile women and promote policy development for the mental health of bereaved women.

## 1. Introduction

Estimates suggest that approximately 48 million couples and 186 million individuals of reproductive age are living with infertility worldwide [1]. Several assisted reproductive technologies (ART) are available for treatment, such as in vitro fertilization (IVF) and intracytoplasmic injection of spermatozoa. However, different factors are decisive for predicting treatment success, such as the woman’s age, duration of infertility, type of infertility, sperm quality, treatment method, number of retrieved oocytes, number of transferred embryos, and embryo quality [2,3].

Although ARTs have allowed infertile couples to achieve viable pregnancies, implantation failure sometimes occurs, and women receiving the treatment can experience consecutive pregnancy losses [4]. Evidence suggests that abortion, fetal death, or even reproductive treatment failure represent a source of suffering that could have a devastating effect on the psychological wellbeing of infertile couples [5,6,7,8] and could lead to prolonged grief responses among women and their partners [7,9].

Deep sadness, depressed mood, irritability, worry, anxiety, and changes in eating and sleeping patterns are considered a part of a normal grief response [10]. Although grief is a natural, nonpathological phenomenon, it can lead to complicated grief reactions, where symptoms are more disruptive, pervasive, or long-lasting than those of normal grief [11]. Complicated grief is a chronic, impairing form of grief resulting from interference with the healing process [12]. This evokes exacerbated reactions, whose symptoms affect the quality of life and can lead to substance abuse and increased risk of suicide [10,11,12,13,14,15]. Furthermore, the individual may feel that life is meaningless, feel confused about their own identity or role in their lives, have difficulties in defining future goals, and feel like dying [11,12,13,14,15].

The distinction between complicated and normal grief occurs especially in the assessment of the intensity and duration of symptoms. Some factors, such as the quality of relationships, the subject’s life history, their historical–cultural nature, and their personality type, directly influence the way grief will be experienced. Among the social factors that contribute to complicated grief are situations where the loss is not talked about. This silencing often occurs in types of grief that are socially invalidated. Therefore, to qualify complicated grief, a longitudinal assessment of the bereaved person is necessary to obtain reliable variables for diagnosis [13,14,15].

Although these feelings are reported as a response to grief, and many studies report grief in response to perinatal loss [16,17,18,19,20], little is known about the positive and negative psychosocial aspects of gestational grief in women experiencing infertility and undergoing treatment. Therefore, our objective was to systematically review and evaluate these psychosocial aspects of gestational grief. Our findings may be valuable in developing strategies to support and promote wellbeing in couples dealing with the involuntary absence of children, both from a psychological and biological perspective for treatment, as well as in the identification of grief. Moreover, this study may contribute to developing strategies that promote and impact adherence to infertility treatment, stress that can have a detrimental impact on the reproductive system, the silence around pregnancy loss, and trauma [15].

## 2. Materials and Methods

We performed a systematic review on the psychosocial aspects of gestational grief in women undergoing infertility treatment. The review was reported in accordance with the recommendations Preferred Reporting Items for Systematic Reviews and Meta-Analyses—PRISMA [21] and was registered in the International Prospective Register of Systematic Reviews (PROSPERO CRD42021233503).

### 2.1. Search Strategy

A researcher defined the search strategies in six databases: MEDLINE/PubMed, EMBASE, CINAHL, Scopus, ScienceDirect, and Lilacs. For our review, we considered articles published up to 5 March 2021. Google Scholar and the reference lists of eligible studies were also analyzed.

To guide the search for relevant studies, a strategy with the acronym PECO was developed. The acronym PECO focuses on population (P), exposure (E), comparison (C), and outcomes (O) [22]. Thus, P = women; E = infertility and infertility treatment; C = fertile women or without comparison groups; and O = psychosocial aspects of grief.

To search for articles in the databases, the following keywords and Medical Subject Headings (MeSH) terms were used, along with the Boolean operators AND and OR:

1. Psychosocial aspects.
2. Grief [MeSH term] OR mourning OR mourning.
3. Reproductive techniques, assisted [MeSH term] OR infertility [MeSH term] OR infertile couple.

The search strategies for each database are presented in Supplementary Table S1.

### 2.2. Inclusion Criteria

The inclusion criteria were (1) studies on the psychosocial aspects of gestational grief among women who experienced the infertility treatment process; (2) studies published up to 5 March 2021; (3) no language restrictions; (4) qualitative or quantitative observational studies. Expert opinion articles, case reports, reviews, and unpublished data were excluded. Studies that addressed the psychosocial responses of parents of babies diagnosed with life-threatening prenatal care, subfertility in women with mental disabilities, infertility in women with cancer, and studies on therapeutic interventions were also excluded.

The terms psychosocial factors, grief, and infertility were defined as follows:

(1) Psychosocial factors: characteristics or facets that influence an individual psychologically and/or socially and act between the social and the individual level. Such factors can describe individuals in relation to their social environment and how these affect physical and mental health [23,24].
(2) Grief: a set of emotional and affective responses related to loss. These reactions are multifactorial and involve cognitive, behavioral, and social aspects [25,26]. Although grief is an expected response to loss, some people may experience prolonged grief. The Diagnostic and Statistical Manual of Mental Disorders (DSM–5) describes the occurrence of a persistent and generalized grief response characterized by persistent yearning or yearning and/or concern for the deceased. The persistent grief response may be accompanied by at least three of eight additional symptoms that include disbelief, intense emotional pain, sense of identity confusion, avoidance of reminders of loss, numbness, intense loneliness, lack of meaning, or difficulty with engaging in ongoing life [15].
(3) Infertility: can be defined as primary or secondary. Primary infertility is when a woman is unable to conceive a child, either due to the inability to become pregnant or carry a pregnancy to term (miscarriage or stillbirth). Secondary infertility is when a woman is unable to conceive a child, either due to the inability to become pregnant or carry a pregnancy to term after a previous pregnancy [27]. Our definition includes the parameter of attempted conception for at least 12 months in women <35 years of age and at least six months in women ≥35 years of age [28].
(4) Gestational grief: grief experienced after pregnancy loss or failure to become pregnant, that is, women who undergo assisted reproduction treatment without being able to become pregnant, and from the gestational loss after assisted reproduction [29,30].

The post-assisted reproduction gestational mourning process may be accompanied by intense anxiety, as well as feelings of guilt, injustice, worthlessness and incapacity, loss of control, and fear. Such conditions need to be evaluated so that grief is prevented or treated [30,31].

### 2.3. Study Selection and Inclusion Process

We performed a two-step selection and inclusion process. First, three independent reviewers (M.H.M.C., F.S.A.T., and C.R.M) examined the titles and abstracts of potentially relevant articles. In the second stage, we obtained the complete articles of the abstracts that were selected and evaluated them in relation to our inclusion criteria. Any disagreements were resolved by discussion with a third reviewer (W.N.A.). The article screening steps were conducted using Rayyan software [32].

### 2.4. Data Extraction

After articles were selected, we discarded duplicate data using Rayyan software [33]. The following information was collected: author, year of publication, place of study, study design, and information on the psychosocial aspects of gestational grief.

### 2.5. Quality Assessment

The quality of cross-sectional studies was analyzed using the Grading of Recommendations, Assessment, Development, and Evaluations (GRADE) [34]. The quality of the study evidence was classified into four categories: high, moderate, low, or very low [35].

The quality of qualitative studies was assessed using the checklist developed by Dixon-Woods et al. [35]. This assessment was based on clarity, consideration of ethical issues, and sample data. In addition, the critical evaluation of the articles was based on 10 questions about the clarity, methods, and results of the studies [34]. Thus, studies were rated as low (one star: 0–3 points), medium (two stars: 4–7 points), or high quality (three stars: 8–10 points) [36].

### 2.6. Statistical Analysis

We summarized the data using descriptive statistics, using frequencies and percentages where relevant, representing how many articles reported a particular outcome measure. All analyses and figures were produced using STATA software version 16.0 (StataCorp, College Station, TX, USA).

## 3. Results

### 3.1. Selection of Studies

**Figure 1:**
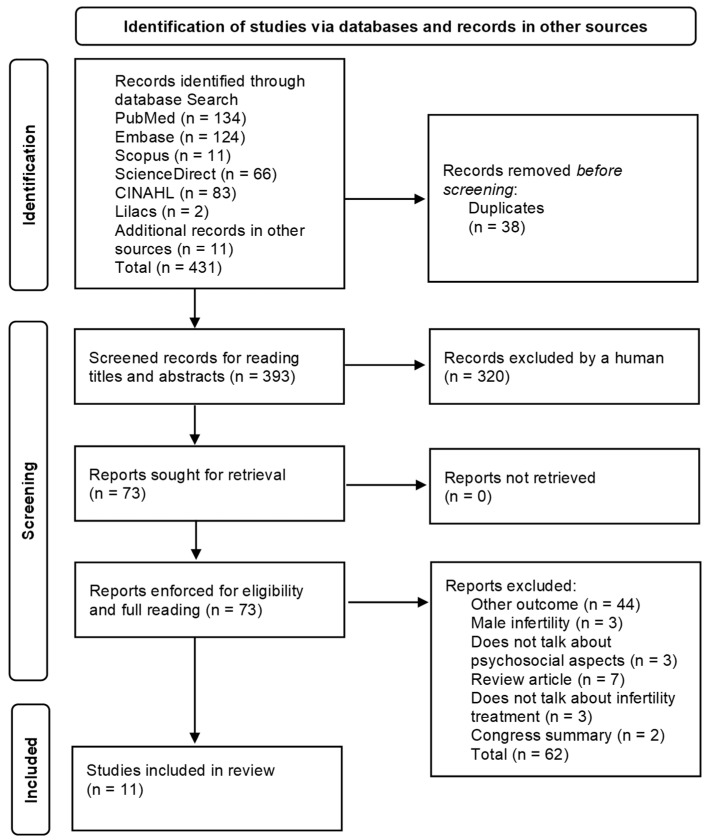
Study selection PRISMA flow diagram.

Our search strategy identified a total of 431 articles; after removing duplicates, 393 titles and abstracts were reviewed, and we selected 73 full-text articles for a full reading. Of these, 11 articles were finally included in the systematic review [6,7,29,30,31,37,38,39,40,41,42] (Figure 1). A flow diagram summarizing the study selection process and reasons for exclusions are shown in Figure 1.

The characteristics of the included studies are shown in Table 1. Three cross-sectional studies [7,30,37] and eight qualitative studies were included. Altogether, the studies included 316 women experiencing infertility, aged between 21 and 55 years. Of these, 214 women received IVF treatment or medication to induce ovulation but were unable to conceive [7,29,30,37]. One hundred and two women became pregnant after fertilization procedures; however, there was a loss of fertilized eggs (embryos) after the IVF treatment, or spontaneous abortion or fetal death occurred [6,32,38,39,40,41,42]. Most studies were published in North America (5/11, 45.45%) and Europe (3/11, 27.27%). There were also publications from Asia (1/11, 9.09%), Oceania (1/11, 9.09%), and the Middle East (1/11, 9.09%).

### 3.2. Psychosocial Aspects

Table 2 presents the psychosocial aspects of grief reported in the quantitative observational studies, and Table 3 presents the results of the qualitative studies. These studies showed both positive and negative aspects of gestational grief.

Five studies presented information about the positive and negative responses to gestational grief [7,32,38,39,40,41]. We identified a relationship between the negative and positive manifestations. The positive manifestations emerged as coping strategies and were correlated with grief responses, which included being self-reliant, fatalistic, supportive, evasive, palliative, and emotive [7].

Interestingly, many women described going through phases of grief, loss, and coping with internal pain with a phase of hope of a new pregnancy or the possibility of becoming pregnant, thus experiencing an apparently infinite cycle with the involuntary absence of motherhood [32,38,41]. However, for women undergoing infertility treatment, the possibility of another pregnancy is uncertain, which causes psychological distress [40].

### 3.3. Positive Psychosocial Aspects

**Figure 2:**
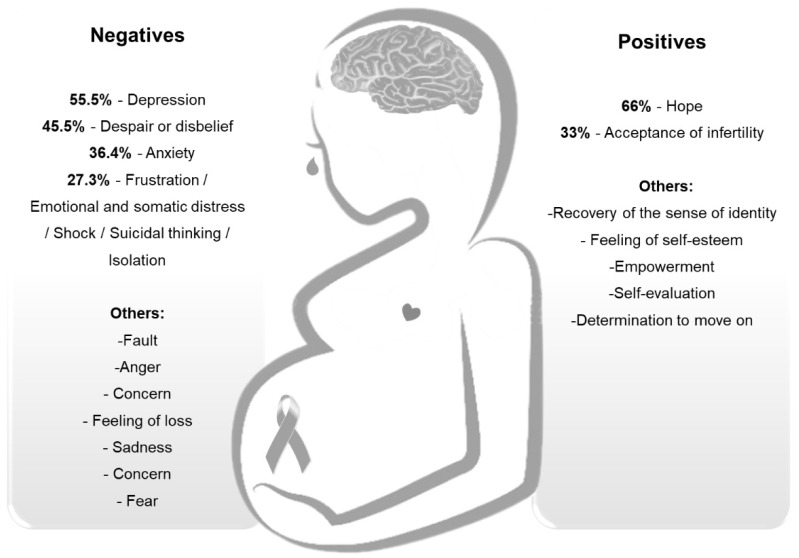
Negative and positive psychosocial aspects of grief in women experiencing infertility undergoing infertility treatment.

The positive aspects of gestational grief were reported in only six studies, and the highest frequency was the hope of becoming pregnant through infertility treatment (4/6, 66.6%) and acceptance of infertility after the infertility treatment attempt (2/6, 33.3%; Figure 2) [7,30,36,37,38,39].

### 3.4. Negative Psychosocial Aspects

The negative psychosocial responses to grief that were most frequently reported included depression (6/11, 54.5%), despair or loss of hope/guilt/anger (5/11, 45.5%), anxiety (4/11, 36.4%), frustration (3/11, 27.3%), and anguish/shock/suicidal thoughts/isolation (2/11, 18.2%; Figure 2).

Furthermore, in the study by Hasanpoor-Azghdy et al. [6], a woman expressed her feelings of grief as follows:

*“When a lot of enthusiasm results in failure, you become extremely hopeless and disenchanted…”.*

*“Sometimes I feel really frustrated, I like to commit suicide”.*

Another patient reported feelings of grief and emptiness as a consequence of not being able to become pregnant [42]:

*“… grief … it is a deep grief … yes, that is perhaps the strongest word that comes directly… emptiness … yes, the meaningfulness … there is something missing in your life … it maybe that … the child part so to say, … it feels as if you have been cheated …”.*

Another feeling described was sadness when receiving the news of the loss of fertilized eggs (embryos) after IVF. The news had an impact on the patient’s emotional and mental state [37]:

*“… the third time, I was pregnant but then, erm … when we went to have the scan, it was really sad, because that … erm … the little embryo, the fetus had died …”.*

### 3.5. Quality Assessment

The results of the quality assessment of the studies are presented in Table 1. The assessment using the GRADE tool indicated that the three observational studies had moderate quality [7,30,37]. The details of the methodology of cross-sectional studies were well reported and there are no serious limitations or inconsistencies. All three studies had a low sample size, so further research is likely to have a major impact on our confidence in the effect estimate and may change the quality assessment estimate. The authors of these studies reported no conflicts of interest in performing the research, and the studies were all approved by the relevant Ethics Committees.

As for the qualitative studies, five had moderate quality [6,32,38,39,42], while three had low quality [30,40,41]. Overall, the eight qualitative studies presented a well-structured methodology but had a small sample size. Therefore, the quality of this evidence does not allow us to assume that the results reflect the true findings of the psychosocial aspects of gestational grief in women undergoing infertility treatment. Thus, we emphasize the importance of carrying out new qualitative studies on this topic. Moreover, five studies reported approval by the relevant ethics committee [6,32,38,41,42], and three studies reported no conflicts of interest [6,38,39].

## 4. Discussion

The objective of this systematic review of the literature was to analyze the psychosocial aspects of gestational grief in women who have undergone infertility treatment. We considered both negative and positive psychosocial aspects in response to grief. Data were obtained from 11 existing studies that collectively studied 316 women experiencing infertility and receiving ART treatment. The results of our review indicate that women experiencing infertility or seeking treatment for infertility may present negative psychosocial responses to grief. The most frequently reported negative responses were depression, despair, or loss of hope in becoming pregnant and having a child, guilt for not being able to become pregnant, anger, anxiety, and frustration due to treatment failure, and anguish, shock, suicidal thoughts, and isolation. Among the psychosocial responses to grief, depression was the symptom that most manifested itself among women experiencing infertility [6,7,30,37,38,39].

Grief in infertile couples is considered a physiological and instinctive response because of the losses experienced due to the absence of children, whether real (for example, failed treatment cycles, miscarriages, or fetal death) or symbolic (for example, never becoming pregnant and consequently losing the sense of identity as a parent) [29,43]. However, considering the differential diagnosis of reactions to grief, it is important to differentiate between the expected reactions, which are transient and inherent to the grief experienced, and the presence of pathological grief. The latter may include major depressive episodes with marked vegetative symptoms, suicide ideation, cognitive impairment, anhedonia, or psychotic symptoms (e.g., excessive punitive thoughts and paranoid delusions) during and after infertility treatment [28,43].

Although the negative psychosocial aspects reported here indicate intense psychological suffering, some women reported positive psychosocial aspects, including the hope of becoming pregnant with infertility treatment and acceptance of infertility after the fertilization attempt [7,38,39,41]. Many of these women have experienced a cycle of hope and empowerment at the time of ovulation and IVF, despair or hopelessness at the failure of treatment, recovery, and a return to hope with each menstrual cycle [38,39,40,41].

Hope may be an important psychosocial aspect in reducing psychological symptoms and facilitating psychological adjustment in infertile couples [44]. Spiritual resources, sympathy, and family support have also been considered a source of hope for women undergoing infertility treatment [45]. In addition, conducting psychological counseling sessions (hope therapy) during reproductive cycles is considered important during treatment [44].

### 4.1. Strengths and Limitations

We highlight several strengths of this review. First, this is the first systematic review to address the psychosocial aspects of grief in women experiencing infertility and undergoing infertility treatment. Second, a systematic review protocol was previously registered. Third, we applied a comprehensive search strategy, with no restriction on the year of publication, performed in six databases of the health sciences. We also assessed the methodological quality of all the published studies. However, some limitations need to be highlighted as well. First, due to the characteristics of the studies included, conducting a meta-analysis was not feasible. Second, most studies had moderate to low methodological quality, mainly due to the small sample sizes of the studies. Lastly, some studies did not report on ethics committee approval or possible conflicts of interest.

### 4.2. Implications for Clinical Practice and Future Research

It is known from existing research that both an infertility diagnosis and the assisted human reproduction treatment process have an impact on the emotional and psychological wellbeing of couples. The treatment represents the hope of being able to have a biological child; however, when the treatment is unsuccessful, and repetitive cycles of loss occur, women and their partners may have negative psychosocial responses to grief. The negative and positive psychosocial aspects of responses to grief presented in this study reinforce the need for psychosocial care for women experiencing infertility grief before, during, and after treatment. This can have significant impacts on their short- and long-term psychological recovery [46].

Cognitive and emotional impacts, including sadness after infertility treatment, are linked to maternal–fetal attachment, that is, the emotional bond that an expectant mother develops with her unborn baby, as described by Sacchi et al. [47]. This maternal–fetus relationship established in the prenatal period is associated with its psychological function, which reinforces the importance of psychological follow-up before, during, and after failures in infertility treatment. Additionally, it is important to diagnose pathological grief quickly and correctly. Further, it is essential that women experiencing infertility undergoing infertility treatment have psychological support and support from their partners, who often invalidate the pain these women experience. It is also important that health professionals are prepared to face this problem.

The quality of the evidence found supports the findings that the assisted human re-production treatment process and infertility have an impact on women’s emotional and psychological wellbeing. It is important to identify and treat women with normal or complicated grief during infertility treatment for the wellbeing of couples from both a psychological and biological perspective. From a psychological perspective, women who experience complicated grief may be less likely to continue trying to become pregnant by adhering to infertility treatment. From a biological perspective, it may be that the stress women are experiencing as a part of grief can lead to the production of stress hormones that can have a detrimental impact on the reproductive system, leading to subsequent pregnancy failure or loss [15,48]. However, we emphasize the importance of new and robust quantitative and qualitative research on the psychosocial aspects of gestational grief, including the assessment of the presence of anxiety and depression, as well as other psychosocial manifestations in assisted human reproduction. Furthermore, studies with results can be compared to a control group.

## 5. Conclusions

In conclusion, the negative psychosocial manifestations of response to grief were depression, despair or loss of hope, guilt, anger, anxiety, frustration, anguish, shock, suicidal thinking, and isolation. The most frequently reported positive manifestations were the hope of becoming pregnant and acceptance of infertility after attempting infertility treatment. Therefore, psychological support before, during, and after assisted human reproduction treatment is crucial for managing psychosocial aspects of responses to grief in infertile couples, as women who seek alternatives to becoming pregnant strive for a future pregnancy as a resolution of their grief. Despite advances in ARTs, the prenatal period still carries many mysteries. It is natural for a woman to expect a spontaneous pregnancy to occur, especially when planned. However, when uncertainties arise regarding the reproductive process, the psychosocial aspects begin to change, which can lead to mental illness among women experiencing infertility. This, once again, confirms the relevance of this study and the importance of a multidisciplinary follow-up period as a support for the processes of coping with grief and pain. Finally, our study can help raise awareness of the area of grief among infertile women and promote policy development for the physical and mental health of bereaved women.

## Figures and Tables

**Table 1:** Characteristics and quality assessment of included studies.

Author and (Year)	Location/Country	Type of Study and Questionnaire	Sample Size	Control Group	Sample Type	Infertility Treatments	Quality of Evidence	Conflict of Interest	Ethical Approval
Quantitative Observational Studies									
Baram et al. (1988)	New York, United States	Retrospective cross-sectional study; Own questionnaire	*n* = 47	N/A	Women who had infertility treatment failure	IVF	⨁⨁⨁◯ MODERATE	NR	NR
Lee et al. (2010)	Taiwan, China	Cross-sectional study; Grief responses questionnaire	*n* = 66	N/A	Women who had infertility treatment failure	IVF	⨁⨁⨁◯ MODERATE	NR	NR
Lukse & Vacc (1999)	North Carolina, United States	Cross-sectional study; Grief Experience Inventory; Depression Adjective Checklist; and Ways of Coping Checklist	*n* = 100	N/A	Women being treated for infertility	IVF (*n* = 50) and medication for ovulation induction (*n* = 50)	⨁⨁⨁◯ MODERATE	NR	NR
Qualitative Studies									
Bell et al. (2013)	New South Wales, Australia	Qualitative research with an interview based on phenomenology	*n* = 28	N/A	Women who had infertility treatment failure	IVF	**	No	Yes
Fieldsend & Smith (2020)	London, United Kingdom	Qualitative research with interpretive phenomenological analysis and interview	*n* = 11	N/A	Women being treated for infertility	IVF	**	No	NR
Freda et al. (2003)	NY, United States	Qualitative research with interview based on phenomenology	*n* = 8	N/A	Women who had infertility treatment failure	IVF, IUI	*	NR	NR
Greenfeld & Decherney (1988)	New Haven, United States	Qualitative research with semistructured psychosocial interview	*n* = 3	N/A	Women who had infertility treatment failure	IVF	*	NR	NR
Hasanpoor-Azghdy et al. (2014)	Tehran, Iran	Qualitative study with interviews conducted using qualitative content analysis	*n* = 25	N/A	Women who had infertility treatment failure	M, IUI e IVF	**	No	Sim
Harris & Daniluk (2010)	Ontario, Canada	Qualitative and phenomenological study with in-depth interviews, recorded on tape	*n* = 10	N/A	Women who had infertility treatment failure	Medication, IUI, IVF, FET	**	NR	Yes
Johansson & Berg (2005)	Goteborg, Suécia	Qualitative research with interview	*n* = 8	N/A	Women who had infertility treatment failure	IVF	*	NR	Yes
Volgsten et al. (2010)	Uppsala, Sweden	Qualitative research with individual semistructured interviews with content analysis	*n* = 10	N/A	Women who had infertility treatment failure	IVF	**	NR	Yes

NR, not reported; N/A, not applicable; M, medicinal; IUI, intrauterine insemination; IVF, in vitro fertilization; FET, frozen embryo transfer; GRADE, Grading of Recommendations, Assessment, Development and Evaluations; ⨁◯◯◯ a filled circle, very low quality; ⨁⨁◯◯ two filled circles, poor quality; ⨁⨁⨁◯ three filled circles, moderate quality; ⨁⨁⨁⨁ four filled circles, high quality. Qualitative studies were rated low (* one star: 0–3 points), medium (** two stars: 4–7 points), and high quality (*** three stars: 8–10 points).

**Table 2:** Psychosocial aspects of grief in women undergoing infertility treatment: quantitative studies.

Author (Year)	Psychosocial Aspects		
	Negatives Mean ± SD		Positive Mean ± SD
Lee et al. (2010)	Responses to grief: Bargain: 3.70 ± 0.64 Depression: 3.14 ± 0.66 Anger: 3.11 ± 0.62 Denial/isolation: 2.98 ± 0.56		Coping strategies: Confrontation: 1.46 ± 0.45 Optimistic: 1.46 ± 0.43 Self-sufficient: 1.42 ± 0.48 Fatalistic: 1.17 ± 0.51 Support: 1.15 ± 0.49 Evasive: 0.97 ± 0.45 Palliative: 0.89 ± 0.36 Emotive: 0.81 ± 0.37 Acceptance: 3.57 ± 0.50
Baram et al. (1988)	Depression: 66% Anxiety: 25.5% Depression and anxiety: 94% Sadness: 85.0% Hopelessness: 51.0% Feeling of loss: 42.5% Guilt / self-censorship: 38.0% Feeling out of control: 34.0% Hypersomnia: 21.0% Insomnia: 15.0% Inability to concentrate: 19.0% Increased appetite: 8.5% Decreased appetite: 6.0% Memory loss: 6.0% Nightmares:10.5% Panic Attacks: 10.5% Suicidal ideation: 13.0% Decrease in performance at work: 15.0%		NR
Lukse & Vacc(1999)	In vitro fertilization Depression: 36% Anger: 3.74 ± 2.21 Denial: 0.84 ± 1.09 Social desire: 2.52 ± 1.71 Despair: 3.70 ± 2.62 Isolation: 1.90 ± 1.74 Loss of control: 2.98 ± 1.61 Dependency: 1.98 ± 1.35	Ovulation induction Depression: 36% Anger: 3.68 ± 2.15 Denial: 0.70 ± 1.02 Social desire: 2.44 ± 1.05 Despair: 3.94 ± 2.26 Isolation: 1.84 ± 1.35 Loss of control: 2.98 ± 1.61 Dependency: 2.04 ± 1.03	NR

NR: not reported.

**Table 3:** Psychosocial aspects of grief in women undergoing infertility treatment: qualitative studies.

Author (Year)	Psychosocial Aspects	
	Negatives	Positive
Greenfeld & Decherney (1988)	Feelings of self-guilt and guilt, anger, emotional and somatic anguish, worry and hostility for not becoming pregnant even after the treatment.	NR
Bell et al. (2013)	Anger and frustration at not being able to conceive naturally. Shock, disbelief, cyclic pattern of grief, loss and disappointment. Depression. Anger and injustice at other people with children, especially those they perceived to be ungrateful or unkind to their children. “complicated grief and loss”.	Cycle of hope and empowerment, despair, repair, and back to hope in each menstrual cycle. Acceptance of infertility after attempted infertility treatment.
Fieldsend & Smith (2020)	Prolonged grief, pain associated with failed infertility treatment and devastation upon learning of embryo death, sense of loss, hidden feeling of loss, sadness, worry, fear, anxiety, depression, emotional distress, anguish and hopelessness. Grief for nonfatal loss.	Hope in attempting infertility treatment, Infighting Self-evaluation Determination to move forward. Positive transformation within you. Recover a sense of identity.
Freda et al. (2003)	Grief and disbelief. Anger/frustration, lack of understanding from others, feeling guilty, feeling lonely/numb with your pain, and gaining strength from adversity. Desperate and helpless about their own fertility. Hopeless. Adoption was considered an admission of defeat. Disbelief.	The inner struggle between hope and hopelessness for future fertility.
Hasanpoor-Azghdy et al. (2014)	Psychological consequences of infertility and medical interventions: psychological turmoil, fear and anxiety and worry, grief and depression. Mental involvement, loneliness, guilt, and regret were only reported as the consequences of infertility. Difficulty in self-control, reduced self-esteem, feelings of failure and helplessness, and hopelessness were experienced after the treatment process.	NR
Harris & Daniluk (2010)	Anguish heightened by pressure from family members to try to become pregnant and insensitive comments that reduced the extent of the losses. Shock, disbelief, and a deep sense of despair and sadness at losing the pregnancy. The women described feeling “completely broken”, “devastated”, “hitting rock bottom”, and “emotionally broken” by the impossibility of ever fulfilling their dreams of becoming a mother. Deep grief, regardless of the baby’s gestational age at the time of the loss. For some, grief was aggravated by multiple pregnancy losses. Feeling of helplessness. In addition to all the losses associated with infertility, for some of these women, miscarriage was a “breaking point” in their efforts to become pregnant. Continuing sadness and loss around the anniversary of their birth date, being acutely aware of the age of the baby they lost had they not miscarried. Feelings of suffering in silence and isolation.	Happiness knowing that they were pregnant.
Johansson & Berg (2005)	Pain for not having children, feelings of incapacity, sadness, feelings of emptiness with the miscarriage.	The treatment gave them a strong sense of self-worth. Feelings of hope for a pregnancy regardless of any known reason for infertility, especially at the time of ovulation.
Volgsten et al. (2010)	Unresolved grief after unsuccessful IFV. Suicidal thoughts, sexual problems, frustration, emptiness, feelings of guilt and disappointment.	NR

NR: not reported; IVF, in vitro fertilization.

## Supplementary Materials

The following are available online at https://www.mdpi.com/article/10.3390/ijerph182413143/s1, Table S1: Estratégias de busca apresentadas nas bases de dados.
