# Supplementary material for: Psychosocial Aspects of Gestational Grief in Women Undergoing Infertility Treatment: A Systematic Review of Qualitative and Quantitative Evidence
Source: Int J Environ Res Public Health. 2021 Dec 13;18(24):13143. doi: 10.3390/ijerph182413143 (PMC8701103; doi:10.3390/ijerph182413143)
Supplement: Supplementary file 1 [file ijerph-18-13143-s001.zip › ijerph-1418746-supplementary.pdf]

Supplementary Table S1. Estratégias de busca apresentadas nas bases de dados.

Databases	Search Strategy
Medline/PubMed	Search: ((psychosocial aspects) AND (grief [MeSH term] OR mourning OR mournings)) AND (reproductive techniques, assisted [MeSH term] OR infertility [MeSH term] OR infertile couple)<br>**Total: 134**
CINAHL	Boolean/Phrase: 1. mourning or grief<br>2. infertility or infertile couple<br>**Total: 83**
EMBASE	1 grief/ 12091<br>2 mourning/1504<br>Advanced<br>3 1 or 2 13239<br>4 female infertility/ or infertility/ or infertile couple.mp. 77690<br>5 3 and 4 124<br>**Total: 124**
Scopus	TITLE-ABS-KEY ((grief OR mourning OR mournings) AND (reproductive AND techniques, AND assisted OR infertility OR infertile AND couple))<br>**Total: 11**
ScienceDirect	((psychosocial aspects) AND (grief [MeSH term] OR mourning OR mournings)) AND (reproductive techniques, assisted [MeSH term] OR infertility [MeSH term] OR infertile couple)<br>**Total: 66**
Lilacs	tw= mourning [Palavras] AND tw= infertility [Palavras]<br>**Total: 2**
